# Neuropsychiatric symptoms as the main determinant of caregiver burden in Alzheimer’s disease

**DOI:** 10.1590/S1980-57642011DN05030008

**Published:** 2011

**Authors:** Renata Kochhann, Ericksen Borba, Maria Otília Cerveira, Diego Onyszko, Alyne de Jesus, Letícia Forster, Luisa Franciscatto, Cláudia Godinho, Ana Luiza Camozzato, Márcia Lorena F. Chaves

**Affiliations:** 1 Dementia Clinic, Neurology Service, Hospital de Clínicas de Porto Alegre, Porto Alegre RS, Brazil; 2 Medical Sciences Post-Graduation Course, UFRGS School of Medicine, Porto Alegre RS, Brazil; 3 Internal Medicine Department and Health Sciences Post-Graduation Course, UFCSPA School of Medicine, Porto Alegre RS, Brazil; 4 Internal Medicine Department, UFRGS School of Medicine, Porto Alegre RS, Brazil

**Keywords:** neuropsychiatric symptoms, caregiver burden, Alzheimer’s disease patients

## Abstract

Caregiver burden is common in Alzheimer’s disease (AD), decreasing the quality of life among caregivers and patients. Projections of aging and aging-related diseases such as AD in developing countries justify additional data about this issue because people living in these countries have shown similarly high levels of caregiver strain as in the developed world.

**Objective:**

The aim of this study was to analyze the association of AD caregivers’ burden with patients’ neuropsychiatric symptoms (NPS), cognitive status, severity of dementia, functional capacity, caregiver sociodemographic characteristics, and the characteristics of care provided by caregivers.

**Methods:**

A cross-sectional study was conducted in a sample of 39 consecutive AD patients and their primary caregivers. NPS were evaluated using the Neuropsychiatric Inventory (NPI). Severity of dementia was assessed with the Clinical Dementia Rating (CDR) scale. Functional capacity was assessed using the Katz and Lawton scales. The burden level was rated using the Burden Interview (BI). Sociodemographic characteristics of caregivers and the characteristics of care provided by them were evaluated. The Mann-Whitney U-test, Kruskal-Wallis test and Spearman’s rho coefficient were performed.

**Results:**

The BI had a moderate correlation with NPI intensity (rho=0.563), p<001. Female caregivers reported a greater level of burden (p=0.031) than male caregivers. The other variables were not significantly associated to caregiver burden.

**Conclusion:**

NPS were the main determinant of burden in primary caregivers of AD patients. This result underscores the need for prevention and treatment of these symptoms. Sex also had an effect on caregiver burden, but the small male sample in this study precludes the generalization of this finding.

## Abstract (pt)

Sobrecarga no cuidador é comum na doença de Alzheimer (DA), diminuindo a qualidade de vida dos cuidadores e pacientes. As projeções de envelhecimento e doenças relacionadas ao envelhecimento como a DA nos países em desenvolvimento justificam dados adicionais sobre esta questão, porque as pessoas que vivem nestes países têm apresentado níveis semelhantes de sobrecarga no cuidador tão alto quanto no mundo desenvolvido.

**Objetivo:**

O estudo teve como objetivo analisar a associação da sobrecarga em cuidadores de pacientes com DA com sintomas neuropsiquiátricos (NPS) dos pacientes, estatus cognitivo, gravidade da demência, capacidade funcional, características sociodemográficas do cuidador e as características dos cuidados prestados pelos cuidadores.

**Métodos:**

Um estudo transversal foi realizado em uma amostra de 39 pacientes consecutivos de DA e seus cuidadores primários. NPS foram avaliados através do Inventário Neuropsiquiátrico (NPI). A gravidade da demência foi avaliada com a escala Clinical Dementia Rating (CDR). A capacidade funcional foi avaliada com as escalas de Katz e Lawton. O nível de sobrecarga foi avaliada utilizando a escala Burden Interview (BI). Características sociodemográficas dos cuidadores e as características de atendimento prestado por eles foram avaliadas. Mann-Whitney, Kruskal-Wallis e coeficiente rho de Spearman foram calculados.

**Resultados:**

BI apresentou correlação moderada com a intensidade do NPI (rho=0,563), p<001. Cuidadoras mulheres relataram maior nível de sobrecarga (p=0,031) do que cuidadores do sexo masculino. As demais variáveis não foram significativamente associados à sobrecarga do cuidador.

**Conclusão:**

NPS foram o principal determinante da sobrecarga do cuidador de pacientes com DA. Esse resultado reforça a necessidade de prevenção e tratamento desses sintomas. Sexo também teve um efeito sobre a sobrecarga do cuidador, mas a pequena amostra do sexo masculino neste estudo evita a generalização dessa constatação.

Alzheimer’s disease (AD) is stressful for those who acquire it, and also for the caregivers of these patients. Some characteristics have been studied to determine caregiver burden including severity of the dementia, behavioral problems and patient’s activities of daily living; age of caregiver, problem-solving ability and perception of disease by caregivers; financial resources, social support, cognitive and functional impairment of patients.^1^

Studies on the care arrangements for people with dementia living in developing countries have shown that levels of caregiver strain were at least as high as in the developed world.^2^ Therefore, the projections of aging and aging-related diseases such as AD in these regions justify the search for additional data about this issue.

In a previous evaluation by our group, no relationship was found between caregiver demographic variables and caregiver distress (evaluated with the Neuropsychiatric Inventory-Distress scale - NPI-D).^3^

As NPI-D analyzes distress related to specific neuropsychiatric symptoms it was decided to evaluate burden in general (using the Zarit Burden Interview - BI) and to analyze the association between caregiver demographic characteristics and BI. However, we first hypothesized that caregivers from developing countries taking care of patients under the public health system, albeit in a university hospital, would be from lower social classes and exhibit higher levels of burden. We also hypothesized that the following characteristics would be associated with higher levels of burden: being in charge of patients with severe dementia; being a full time caregiver; and having lower educational attainment. Therefore, the aim of the study was to analyze the association of AD caregivers’ burden with patients’ neuropsychiatric symptoms, cognitive status, severity of dementia, functional capacity, caregiver sociodemographic characteristics, and the characteristics of care provided by caregivers.

## Methods

A cross-sectional study was conducted in a sample of 39 consecutive patients and their primary caregivers. Participants were enrolled from the Dementia Clinic of Hospital de Clinicas de Porto Alegre (a public university hospital) after giving informed consent. Primary caregiver was defined as someone who spent at least 20h per week giving care. All patients fulfilled the DSM-IV criteria for dementia,^4^ and the NINCDS-ADRDA criteria for probable Alzheimer’s disease.^5^

For assessment of cognitive status, all patients underwent evaluation with the Mini Mental State Examination (MMSE).^6-8^ Functional capacity was evaluated with the Katz and Lawton scales.^9,10^

The severity of dementia was assessed using the Clinical Dementia Rating (CDR) scale.^11-13^ The CDR evaluates memory, orientation, judgment and problem solving, community affairs, home and hobbies, and personal care. This instrument allows the clinicians to categorize patient disease into mild (CDR=1), moderate (CDR=2) or severe (CDR=3) dementia.

Neuropsychiatric symptoms were evaluated using the Neuropsychiatric Inventory (NPI).^14,15^ The NPI consists of three sections for each symptom: frequency, severity, and distress. The information was obtained from the caregiver who had observed the symptoms in the patient during a specific period. Each caregiver was asked an initial screening question for each of the 12 neuropsychiatric domains. If the screening question was negatively answered, the interviewer moved on to the next domain. If it was positively answered, further questions were asked, allowing scores for both severity (range: 1[mild]–3 [marked]) and frequency (range: 1[occasional]–4[very frequent]) of each symptom. An intensity score was then obtained by multiplying the scores of severity by frequency (range: 0–12 for each item).

Burden was rated with the Burden Interview (BI).^16-18^ BI consists of a 22-item questionnaire which verifies the burden perceived by the caregivers concerning: request for help from patients, lack of personal time, feeling of tiredness, embarrassment, irritation, tension, lack of privacy, feeling that the caregiver’s social life is impaired, among others. Questions from 1 to 21 are answered by assigning the following levels of frequency: never=0, rarely=1, sometimes=2, quite frequently=3, or nearly always=4. Question 22 assesses, in general terms, the burden felt by the caregiver and can be scored using the following alternatives: not at all=0, a little=1, moderately=2, quite a bit=3, extremely=4. Total scores range from 0 to 88, and the higher the score, the higher the level of burden.

Caregivers’ demographic data and care characteristics (i.e., relationship with the patient - spouse, daughter/son, other relative, non relative -, self-reported number of hours per week spent giving care, time as caregiver, whether the caregiver lives with patient and whether the caregiver receives payment for care) were evaluated. Caregiver social class was also evaluated with a Brazilian scale which allows classification into A, B, C, D or E classes, where A is the highest level.^19^

### Statistical analyses

Descriptive statistics (mean, SD and frequency) were calculated for demographic data, NPI scores of intensity, MMSE scores and BI total score. The Mann-Whitney U-test, Kruskal-Wallis test and Spearman’s rho coefficient were performed. The statistical analysis was performed using the Statistical Package for the Social Sciences (SPSS 18.0 for Windows).

## Results

Demographic and clinical data from patients and caregivers are shown in Table 1.

**Table 1:** Clinical and demographic data of patients and caregivers.

Variables	AD patients	Caregivers
Sex N (%)		
Female	26 (67)	31 (80)
Male	13 (33)	8 (20)
CDR N (%)		
1	13 (33)	-
2	13 (33)	-
3	13 (33)	-
Relationship to patient		
Spouse	-	11 (28)
Daughter/son	-	27 (69)
Other relative	-	1 (3)
Social class N (%)		
A and B	-	25 (64)
C, D and E	-	14 (36)
Full time job as caregiver N (%)		
Yes	-	29 (74)
No	-	10 (26)
Caregiver lives with patient N (%)	-	33 (85)
Caregiver is unpaid for care of patient N (%)	-	38 (97)
Time as caregiver in months* (range)	-	62.10±49.68 (7-216)
Caregiver hours per week* (range)	-	100.69±23.38 (25-112)
Age* (range)	77.56±8.01 (52-90)	52.90±13.11 (20-79)
Years of education* (range)	4.54±4.14 (0-16)	10.08±4.79 (2-22)
MMSE* (range)	10.54±6.58 (0-24)	-
NPI Intensity* (range)	33.51±22.56 (0-82)	-
ADL* (range)	8.13±6.14 (0-18)	_
IADL* (range)	10.82±3.42 (3-14)	_
BI* (range)	-	33.51±15.75 (3-68)

* mean±SD

BI presented moderate correlation with NPI intensity (rho=0.563), p<001. We also found similar distribution of NPS intensity among the different stages of dementia (severity) (Kruskal-Wallis U-test) (p=0.132). Other patient and caregiver variables showed no correlation with BI (i.e., age, education, time as caregiver, hours of care per week).

The data for comparison of the BI scores according to category (i.e., sex, dementia severity, social class) is given in Table 2. Female caregivers had higher levels of burden (p=0.031) than male caregivers. No other categorical variable presented a statistically significant effect on caregiver BI scores.

**Table 2:** Analyses of BI scores on categorical variables.

Variables	BI mean±SD	p value
Caregiver's sex*		
Female	36.19±16.11	
Male	23.13±8.93	0.031
Caregiver lives with patient*		
Yes	35.33±15.48	
No	23.50±14.48	0.106
Full time job as caregiver*		
Yes	34.28±15.82	
No	31.30±16.17	0.809
CDR**		
1	33.08±19.64	
2	33.85±11.67	
3	34.62±16.13	0.796
Relationship to patient**		
Spouse	31.73±19.59	
Daughter/son	34.59±14.45	
Other relative	24.00	0.618
Social class*		
A and B	31.92±13.88	
C, D and E	36.36±18.86	0.379
Caregiver's type of care*		
Paid	47.00	
Unpaid	33.16±15.80	0.286

* Mann-Whitney Test;

** Kruskal-Wallis Test.

## Discussion

This study evaluated the association of burden of AD caregivers with patients’ neuropsychiatric symptoms, cognitive status, dementia severity, functional capacity, caregiver sociodemographic characteristics, and characteristics of care provided by caregivers.

The main finding was a positive correlation between burden scores and neuropsychiatric symptoms (NPS), suggesting that behavioral disturbances are a key-factor predisposing caregivers to distress, life disruptions, other physical and mental suffering. Other studies have previously reported this finding.^20-23^ Low levels of informal social support,^20^ decreased patient quality of life,^23^ lower patient functional capability^23^ and severity of cognitive decline^21^ were also associated with higher levels of burden in theses studies.

In a previous study by our group,^3^ a significant correlation between total severity NPI and distress NPI was observed, but none of the caregiver demographic data were shown to be associated with distress. Taken together, previous and current results showed NPS to be correlated to distress and burden, independent of any specific neuropsychiatric or general symptoms. In the first study, apathy was the symptom responsible for the highest distress level, followed by agitation and aggression. The most frequent symptoms were apathy and aberrant motor behavior. Patients’ relatives also considered apathy as the most severe symptom, followed by depression and agitation.^3^

In the present study, neither cognitive status or dementia severity was associated to levels of burden, supporting the notion that cognition and intensity of disease are not determinants of burden. Other patient and caregiver variables also showed no relationship with burden. However, sex had an effect on caregiver burden, but the small size of the male sample (N=8, 20%) prevents generalization of this finding.

Individuals from developing countries taking care of patients, users of the public health system, albeit a university hospital were from lower social classes and had higher levels of burden. Besides lower financial means, inaccessibility to other resources could render these individuals more prone to burden. However, the findings of the present study failed to confirm this relationship. More than half of the sample (64%) pertained to upper classes according to the Brazilian classification, and the variability of burden was high. This Brazilian classification for social class may present a bias towards higher levels because it takes into account purchasing power together with educational attainment, but income is not adequately weighted. Despite being largely used this measure may introduce some degree of bias.

The characteristics of being in charge of patients with severe dementia, being a full time caregiver, and having lower educational attainment were also not associated with higher burden. The distribution of the Burden Interview scores among dementia severity classes of patients was similar as were levels of neuropsychiatric symptoms (NPS) evaluated with the NPI, suggesting caregiver burden and patient intensity of neuropsychiatric symptoms may be independent of stage of disease (severity).

The main objective of the study was to assess primary caregivers and therefore most of the individuals were fulltime carers since this reflects the most common profile in Brazil.^21^ The present study showed a similar caregiver profile to Moscoso’s study.^21^

One limitation of this investigation, besides the small sample size, was the lack of medical and personal variables from the caregiver such as depression, anxiety, and management strategies that could influence the perception of burden. Nevertheless, the objective of the study was the analysis of sociodemographic data, care and dementia-related characteristics.

As neuropsychiatric symptoms are common in dementia and affect virtually all patients at some point in the course of the illness,^24,25^ the prevention and treatment of these symptoms are needed. Concerns about the safety and efficacy of psychotropic medications have been raised. Despite these concerns, medications are often prescribed for neuropsychiatric symptoms.^26-28^ Specific types of caregiver and residential care staff education, behavior management therapies, and possibly cognitive stimulation, appear to offer long-term effectiveness for the management of dementia-associated neuropsychiatric symptoms.^29^
